# NT Pro BNP Plasma Level and Atrial Volume Are Linked to the Severity of Liver Cirrhosis

**DOI:** 10.1371/journal.pone.0068364

**Published:** 2013-08-05

**Authors:** Anna Licata, Salvatore Corrao, Salvatore Petta, Chiara Genco, Mauro Cardillo, Vincenza Calvaruso, Giuseppe Cabibbo, Fatima Massenti, Calogero Cammà, Giuseppe Licata, Antonio Craxì

**Affiliations:** 1 Sezione di Gastroenterologia, Dipartimento Biomedico di Medicina Interna e Specialistica, DI.BI.M.I.S, University of Palermo, Palermo, Italy; 2 Sezione di Medicina Interna e Cardioangiologia, Dipartimento Biomedico di Medicina Interna e Specialistica, DI.BI.M.I.S, University of Palermo, Palermo, Italy; 3 Sezione di Igiene, Dipartimento di Scienze per la Promozione della Salute, University of Palermo, Palermo, Italy; University College London, United Kingdom

## Abstract

**Background and Aims:**

Plasma levels of NT-pro-BNP, a natriuretic peptide precursor, are raised in the presence of fluid retention of cardiac origin and can be used as markers of cardiac dysfunction. Recent studies showed high levels of NT pro BNP in patients with cirrhosis. We assessed NT pro-BNP and other parameters of cardiac dysfunction in patients with cirrhosis, with or without ascites, in order to determine whether the behaviour of NT pro BNP is linked to the stage of liver disease or to secondary cardiac dysfunction.

**Methods:**

Fifty eight consecutive hospitalized patients mostly with viral or NAFLD-related cirrhosis were studied. All underwent abdominal ultrasound and upper GI endoscopy. Cardiac morpho-functional changes were evaluated by echocardiography and NT-pro-BNP plasma levels determined upon admission. Twenty-eight hypertensive patients, without evidence of liver disease served as controls.

**Results:**

Fifty eight cirrhotic patients (72% men) with a median age of 62 years (11% with mild arterial hypertension and 31% with type 2 diabetes) had a normal renal function (mean creatinine 0.9 mg/dl, range 0.7–1.06). As compared to controls, cirrhotic patients had higher NT pro-BNP plasma levels (365.2±365.2 vs 70.8±70.6 pg/ml; p<0.001). Left atrial volume (LAV) (61.8±26.3 vs 43.5±14.1 ml; p = 0.001), and left ventricular ejection fraction (62.7±6.9 vs. 65.5±4%,; p = 0.05) were also altered in cirrhotic patients that in controls. Patients with F2-F3 oesophageal varices as compared to F0/F1, showed higher e' velocity (0.91±0.23 vs 0.66±0.19 m/s, p<0.001), and accordingly a higher E/A ratio (1.21±0.46 vs 0.89±0.33 m/s., p = 0.006).

**Conclusion:**

NT-pro-BNP plasma levels are increased proportionally to the stage of chronic liver disease. Advanced cirrhosis and high NT-pro-BNP levels are significantly associated to increased LAV and to signs of cardiac diastolic dysfunction. NT pro-BNP levels could hence be an useful prognostic indicators of *early decompensation* of cirrhosis.

## Introduction

The foremost hemodynamic feature of cirrhosis is the hyperdynamic syndrome, a circulatory state characterized by low arterial pressure, high cardiac output and decreased peripheral vascular resistance [1]–[3]. As a consequence of this hemodynamic profile, a broad spectrum of cardiac abnormalities, such as impaired myocardial contractility, impaired diastolic relaxation and electrophysiological abnormalities in the absence of any other evident heart disease may be found in patients with cirrhosis [1], [4]–[6]. Both hemodynamic and cardiac profiles denote the pathophysiological scenario known as “cirrhotic cardiomyopathy”, a clinical condition which plays a role in the course of cirrhosis, at a rate whose relevance is yet partly undefined [1], [6]–[7].

During the past few years, considerable attention has been paid to the role of markers of cardiac dysfunction, BNP and its pro-hormone, NT pro BNP, which both are secreted by heart ventricles in response to massive stretching of muscle cells or to mild cardiac damage and show capability to reduce blood pressure and cardiac hypertrophy [8]–[12]. Further, recent studies revealed that high serum levels of NT pro-BNP are present in patients with chronic liver diseases of viral etiology [13]–[16]. As they seem to be relate to the severity of liver disease and cardiac dysfunction they should be useful markers to identify cirrhotic patients with increased cardiovascular risk and thus, worse prognosis.

In this study we aimed to assess in a well-characterized cohort of patients with cirrhosis of non-alcoholic aetiology, before or after the development of ascites, the expression of NT pro-BNP and of other parameters of cardiac dysfunction in order to determine whether the behaviour of NT pro BNP is linked to the stage of liver disease or to a cardiac dysfunction secondary to cirrhosis.

## Patients and Methods

The study was performed in accordance with the principles of the Declaration of Helsinki and its appendices, and with local and national laws. Approval was obtained from the AOUP Policlinico “ Paolo Giaccone” of Palermo, Institutional Review Board and Ethics Committee, and written informed consent was obtained from all patients and controls.

From April 2010 to October 2011, we consecutively enrolled fifty eight patients with a clinical or histological diagnosis of cirrhosis, admitted to our liver Unit, which is a tertiary referral centre for chronic liver disease. All of them underwent, abdominal ultrasound and upper GI endoscopy in order to determine the stage of liver disease and the presence of ascites and indirect signs of portal hypertension.

All patients with known cardiac or renal diseases (creatinine >1,5 mg/dl) were excluded. The diagnosis of arterial hypertension was based on the following criteria: systolic blood pressure ≥135 mm Hg and/or diastolic blood pressure ≥85 mm Hg (measured three times within 30 minutes, in the sitting position and using a brachial sphygmomanometer), or use of blood-pressure-lowering agents. The diagnosis of type 2 diabetes was based on the revised criteria of the American Diabetes Association, using a value of fasting blood glucose ≥126 mg/dL on at least two occasions [17]. In patients with a previous diagnosis of type 2 diabetes, current therapy with insulin or oral hypoglycemic agents was documented.

We also evaluated a control group of 28 newly diagnosed hypertensive patients matched for age and sex and who were referred to Internal Medicine Unit for clinical investigation. All had a normal renal function without any signs of major organic disease, including liver disease. In particular, all were negative for HBsAg, anti-HCV, anti-HIV, and all of them had normal liver function tests (LFTs).

### Echocardiographic assessment

All cirrhotic and control patients underwent echocardiographic examination to study diastolic and systolic cardiac function.

One experienced observer performed all echocardiograms according to the recommendations of the American Society of Echocardiography (a) using a GE VIVID 7 Dimension Cardiac – Vascular Ultrasound, interfaced with a 2.5 MHz phased-array probe. All recordings were digitally stored for off-line analyses by dedicated software.

M-mode echocardiograms of the left ventricle were recorded from the parasternal long-axis view guided by two-dimensional image. We measured left ventricular internal diameter (LVID), interventricular septum (IVS), and posterior wall thickness (PWT) on this M-mode tracings, as described in the American Society of Echocardiography guidelines [18]. When optimal orientation of the M-mode ultrasound beam could not be obtained, we performed linear measurements on correctly oriented two-dimensional images. We determined left atrial (LA) diameter and aortic root (AO) diameter on M-mode images. All the measurements of cavities and wall thickness were made by the leading-edge method (http://www.ncbi.nlm.nih.gov/pubmed/2698218) and averaged over three cardiac cycles. The ejection fraction (EF) was calculated by using the area-length formula as previously described [19]. We computed LV mass in grams using the following formula: 0.8×(1.04[(LVID + IVS + PWT)3− LVID3]) +0.6 [20]. LV mass was corrected by body surface area of each patient. Left atrial end-systolic volumes and Left Ventricular End-Diastolic ones (monoplane area/length from four chamber view, Dodge correction) were computed. From the apical window, the sonographer placed the Doppler sample volume between the tips of the mitral valve to obtain pulsed-wave Doppler images of mitral blood flow. From the transmitral flow signal, he measured peak early diastolic velocity (E) and peak late diastolic velocity (A) and calculated the E/A ratio. The observer acquired tissue Doppler images (TDI) of septal and lateral left ventricle segments. From these recordings, we determined for each segment peak early (e′) TDI velocities and calculated the E′/e′ ratio. For the analysis, we averaged e′ measured from septal and lateral sites.

### Grading diastolic dysfunction

Assessment of diastolic dysfunction, according to Recommendations for the Evaluation of Left Ventricular Diastolic Function by ASE [20] take into consideration mitral E, E/A ratio, e′ and E/e′ ratio. Mild diastolic dysfunction was defined as following reported according to ASE guidelines [21]: mitral E/A ratio <0,8; anular e′<8 cm/s and E/e′ratio <8 (septal and lateral). Moderate diastolic dysfunction was defined as: mitral E/A ratio 0,8–1,5; anular e′ <8 cm/s and E/e′ratio 8–12 (septal and lateral). Severe diastolic dysfunction was defined as: mitral E/A ratio >2; anular e′<8 cm/s and E/e′ratio >13 (septal and lateral).

### Plasma NT-pro-BNP analysis

Plasma NT-pro-BNP levels were determined by a commercial assay (Vitros NT-pro-BNP, Ortho-Clinical Diagnostics, Inc.). Blood samples from cirrhotic and controls have been drained and then centrifuged at 2000 rpm for 15 minutes at room temperature. The supernatant plasma was recovered and stored at −80°C until analysis.

### Statistical Analysis

Continuous variables were summarized as mean ± SD; categorical variables as frequency and percentage. The ANOVA test, student's t-test, Kruskal-Wallis test and chi-square test were used when appropriate.

Univariate linear regression analysis was used to study relationships between NT-pro-BNP and clinical, biochemical and echocardiographic variables of cirrhotic patients.

Logistic regression model was used to identify the factors associated with the presence of ascites, by assessing the same independent variables included in the linear model. A two-tailed p value of p<0.05 was considered significant. STATA/SE, version 12.1 for Windows (Stata Corp, College Station, TX), was used to analyze the data.

## Results

The baseline features of the 58 cirrhotic and 28 control patients are shown in Table S1.

Cirrhotic patients (72% men) had a median age of 62 years, a prevalence of arterial hypertension and diabetes of 11% and 31% respectively, and showed a renal function within the normal range (range 0.7–1.06) with a mean creatinine levels of 0.9 mg/dl. The liver disease was due to HCV infection (58.6%), HBV infection (13.7%), alcohol abuse (3.4%), NASH (13.7%), and cryptogenic liver damage 6 (10.4%). According to Child-Pugh classification, patients were divided as follows: 24 (41.4%) in A class; 23 (39.7%) and 11 (18.9%) in B and C classes, respectively. Further, all 58 patients were subgrouped according to the presence of ascites; 32 out of 58 (55.2%) were cirrhotic decompensated patients being on therapy with diuretics or paracentesis (refractory ascites); the remaining 44.8% were compensated (without ascites). For patients with refractory ascites, echocardiographic assessment was done after paracentesis. Upper GI endoscopy showed that 10 (17.3%) patients did not have signs of portal hypertension, while 24 (41.4%) had small (F1) oesophageal varices and 22 (37.9%) had medium-large (F2/F3) oesophageal varices. Only for two patients type and dimension of esophageal varices were not assessed. Twenty five patients (43.1%) were on beta blockers as prophylaxis of first variceal bleeding or rebleeding. Finally, in 25 (43.1%) cases a diagnosis of HCC was made during hospitalization, but none of these patients was on therapy with anti-angiogenic drugs.

The control subjects (mean age of 60.6±8,4 years, 20 male and 8 female) were comparable for diabetes prevalence to the cirrhotic population, and had similar creatinine serum levels.

### NT proBNP plasma level and echocardiographic parameters

NT pro-BNP plasma levels and echocardiographic parameters of both cirrhotic and control patients are showed in Table 1.

**Table 1:** NT pro BNP serum levels and echocardiographic features of 58 cirrhotic patients and 28 matched controls.

Variable	Cirrhotic Patients (n = 58)	Control Population (n = 28)	p value
NT pro BNP – pg/ml*	365.2±365.2	70.8±70.6	<0.001
**Mean septal/lateral e**'	0.10±0.03	0.11±0.07	0.22
**MV E Vel – m/s**	0.77±0.24	0.69±0.18	0.14
**E/e**' **ratio**	8.2±3.1	7.2±2.5	0.20
**Left Atrium – ml**	61.8±26.3	43.5±14.1	0.001
**MV E/A ratio**	1.07±0.40	1.04±.044	0.70
**LVEDV MOD a4C – ml**	90.3±33.3	79.9±26.7	0.16
**EF – %**	62.7±6.9	65.5±4.0	0.05
**PAPs – mmHg**	30.4±4.9	28.1±1.7	0.08
**LVd Mass Ind (ASE) – g/m2**	84.3±24.0	73.9±18.3	0.08

**Figure 1:**
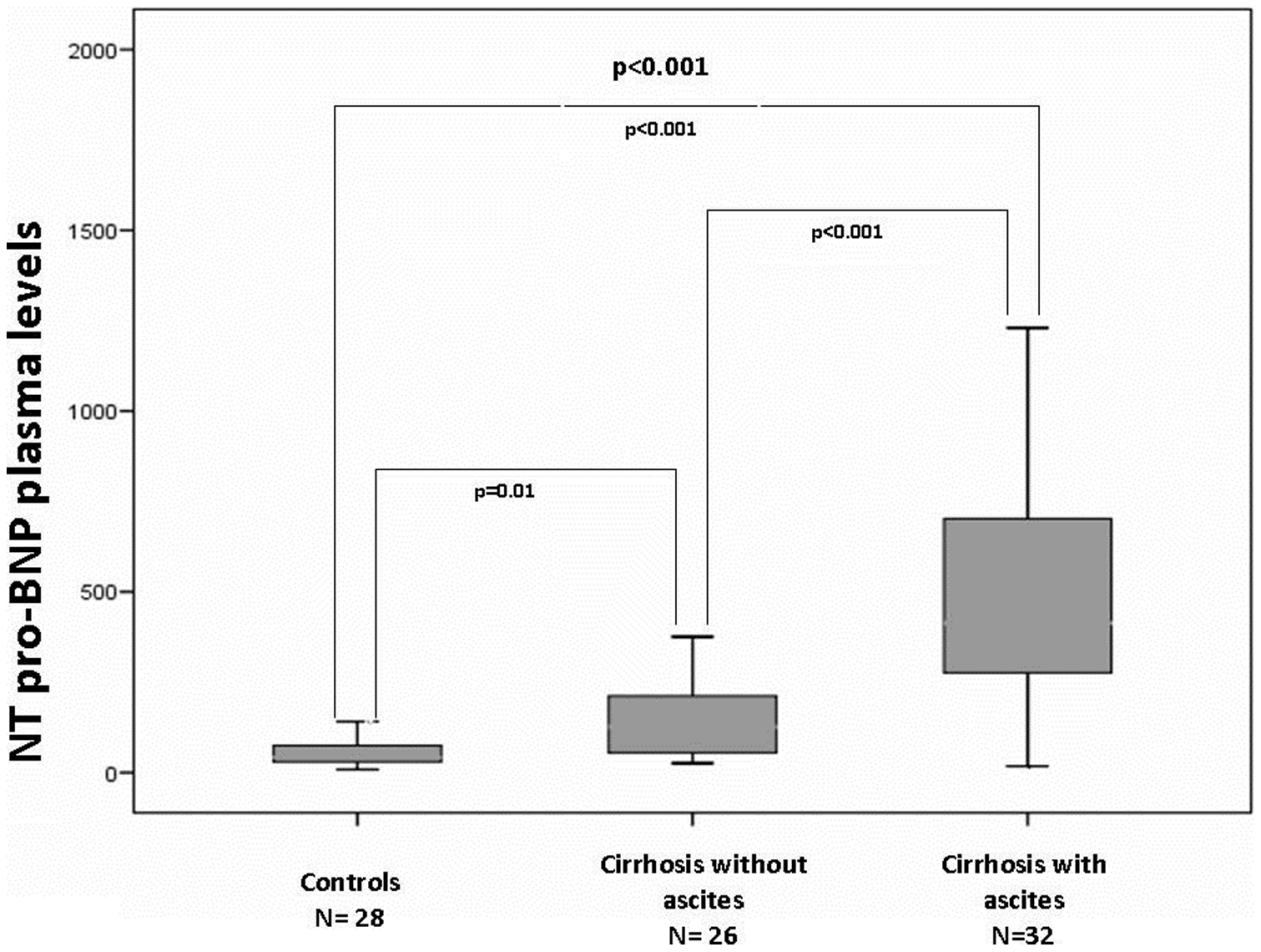
Cirrhotic patients with and without ascites compared to control hypertensive population had significantly higher NT pro-BNP plasma levels.

Cirrhotic patients had significantly higher NT pro-BNP plasma levels compared to controls (365.2±365.2 vs 70.8±70.6 pg/ml; p<0.001) (Figure 1).

Similarly, left atrial volume (LAV) (61.8±26.3 vs 43.5±14.1 ml; p = 0.001), and left ventricular ejection fraction (62.7±6.9 vs. 65.5±4%,; p = 0.05) were significantly altered in cirrhotic patients as compared to controls, and a trend was also observed for left ventricular mass (84.3±24 vs. 73.9±18.3 g/m^2^; p = 0.08) and Systolic Pulmonary Arterial Pressure (PAPs) (30.4±4.9 vs. 28.1±1.7 mmHg; p = 0.08).

### Factors associated with NT pro-BNP plasma levels in cirrhotic patients

By linear regression analysis, in cirrhotic patients, at univariate analysis, NT-pro-BNP levels were directly related to liver dysfunction (lower albumin, lower INR, presence of ascites, cirrhosis stage, higher MELD and Child Pugh scores), and to renal impairment (higher serum creatinine levels), and to larger atrial volumes (Table 2).

**Table 2:** Relationship between NT-pro-BNP levels and clinical and echocardiographic data on linear regression analysis in cirrhotic patients.

	Univariate Analysis		
	β	S.E.	*p* value
**Age**	0.201	0.15	4.566
**Sex**	−0.138	111.388	0.32
**MELD**	0.468	9.436	0.001
**Ascites**	0.486	88.947	<0.001
**Child Pugh**	0.408	22.445	0.002
**Albumine**	−0.333	87.120	0.01
**Bilirubine**	0.192	17.913	0.18
**INR**	0.420	150.649	0.002
**Platelets**	−0.194	0.784	0.17
**Creatinine**	0.349	180.365	0.01
**AST**	−0.145	0.592	0.31
**ALT**	−0.210	0.532	0.14
**Spleen diameter**	0.035	13.671	0.81
**Portal vein diameter**	0.206	19.058	0.18
**HCC**	−0.136	94.038	0.33
**Oesophageal varices**	−0.024	55.798	0.86
**Cirrhosis stage**	0.479	70.308	<0.001
**Arterial Hypertension**	−0.228	113.046	0.10
**Diabetes**	0.033	106.852	0.81
**Beta-blocker therapy**	0.112	102.925	0.42
**Mean e**' **sept/ e**' **lat**	0.059	1623.886	0.70
**E Vel**	0.009	224.385	0.94
**E/e**' **ratio**	0.100	0.001	0.43
**Left atrium Vol (ml)**	0.306	2.395	0.03
**E/A Ratio**	0.170	124.013	0.24
**LEVDV**	−0.008	1.629	0.95
**EF%**	0.123	7.250	0.38
**PAPs**	0.185	12.129	0.28
**Left ventricular mass**	0. 210	2.640	0.89

### NT pro-BNP plasma levels, echocardiographic features and features of portal hypertension

Table 3 shows factors associated with presence of ascites. As expected cirrhotic patients with ascites had a higher impairment of both liver (albumin 3.5±0.5 vs. 2.9±0.4, p<0.001; bilirubin 1.0±0.4 vs. 3.1±3.4, p = 0.005; INR 1.1±0.1 vs. 1.4±0.3, p = 0.002) and kidney function (creatinine 0.7±0.1 vs. 1.0±0.3, p = 0.007), compared to their counterpart without ascites. They also had higher NT pro-BNP plasma levels (171.9±165.9 vs. 525.2±408.1, p<0.001) (Figure 1), and a trend to larger atrial volumes (54.7±21.2 vs. 67.2±28.8 ml, p = 0.09) and to a higher E/A ratio (0.93±0.41 vs. 1.13±0.45, p = 0.08).

**Table 3:** Demographic, clinical, laboratory and echocardiographic features of cirrhotic patients according to presence or absence of ascites.

Variable	Cirrhotic Patients without ascites (n = 26)	Cirrhotic Patients with ascites (n = 32)	p value
**Mean Age – yrs**	63.5±10.9	62.4±10.5	0.69
**Male Gender**	15 (57.7)	27 (84.3)	0.02
**MELD**	5.7±2.9	12.6±3.9	<0.001
**Albumine – g/dl**	3.5±0.5	2.9±0.4	<0.001
**Bilirubin – mg/dl**	1.0±0.4	3.1±3.4	0.005
**INR**	1.1±0.1	1.4±0.3	0.002
**Hepatic Encephalopathy**			
** **Grade 1Grade 2Grade 3	26 (100.0)0 (0.0)0 (0.0)	28 (87.5)4 (12.5)0 (0.0)	0.06
**Platelets – 10^3^/mmc**	98.2±66.6	85.1±63.3	0.46
**Aspartate aminotransferase – IU**	82.9±66.6	73.7±87.8	0.65
**Alanine aminotransferase – IU**	89.3±114.7	46.2±48.9	0.06
**Creatinine – mg/dl**	0.7±0.1	1.0±0.3	0.007
**Spleen Diameter – cm**	14.9±4.8	16.4±2.8	0.18
**Portal vein Diameter – mm**	12.2±1.9	12.8±2.9	0.43
**Oesophageal Varices**			
** **absentF1F2-F3	4 (15.4)13 (50.0)9 (34.6)	6 (20.0)11 (36.7)13 (43.3)	0.58
**Hepatocellular Carcinoma**	12 (46.1)	13 (40.6)	0.74
**Arterial Hypertension**	7 (26.9)	4 (12.5)	0.18
**Type 2 Diabetes**	5 (19.2)	13 (40.6)	0.06
**Beta-blocker therapy**	11 (42.3)	14 (43.7)	0.91
NT pro BNP – pg/ml*	171.9±165.9	525.2±408.1	<0.001
**Mean septal/lateral e**'	0.09±0.03	0.10±0.02	0.19
**MV E Vel – m/s**	0.72±0.20	0.81±0.26	0.19
**E/e**' **ratio**	8.7±3.9	7.8±2.2	0.32
**Left Atrium – ml**	54.7±21.2	67.2±28.8	0.08
**MV E/A ratio**	0.93±0.41	1.13±0.45	0.09
**LVEDV MOD a4C – ml**	82.7±38.9	96.3±27.3	0.13
**EF – %**	61.6±9.0	63.6±4.3	0.29
**PAPs – mmHg**	29.2±4.5	31.5±5.1	0.16
**LVd Mass Ind (ASE) – g/m2**	87.5±24.2	81.2±23.8	0.38

**Figure 2:**
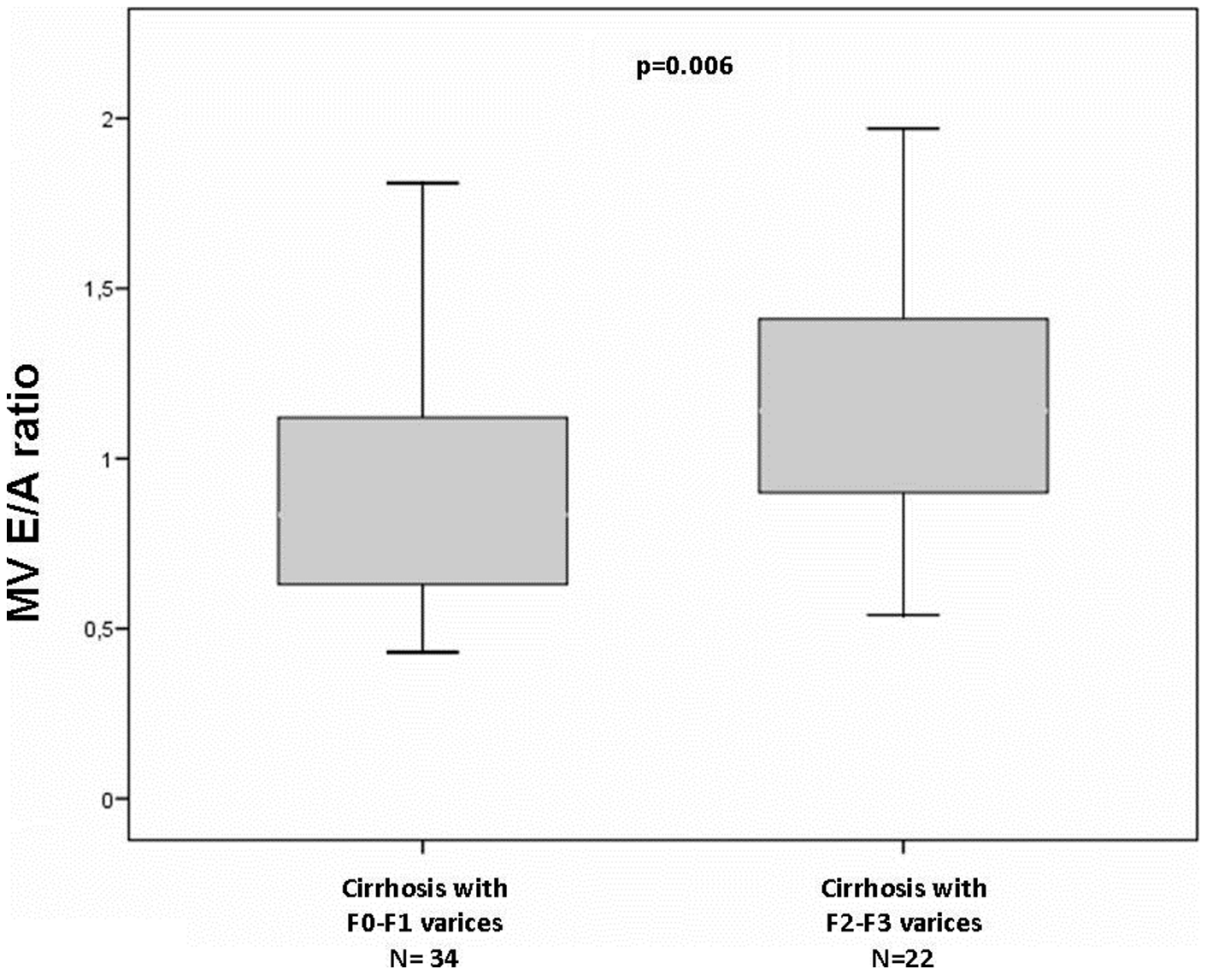
Cirrhotic patients with F2-F3 oesophageal varices compared to F0/F1, even if they had similar NT pro-BNP plasma levels, they showed also a higher E/A ratio as compared to their counterpart.

When comparing patients with F2-F3 oesophageal varices to those with F0/F1 varices (Table 4), regardless of ascites, while NT pro-BNP plasma levels were similar (385.9±329.4, vs. 302.8±338.5 p = 0,39), the former had a higher e' velocity (0.91±0.23 vs 0.66±0.19 m/s, p<0.001), and, accordingly a higher E/A ratio (1.21±0.46 vs 0.89±0.33 m/s., p = 0.006) (Figure 2).

**Table 4:** Demographic, clinical, laboratory and echocardiographic features of cirrhotic patients according to presence or absence of F2-F3 oesophageal varices.

Variable	F0–F1 Varices (n = 34)	F2–F3 Varices (n = 22)	*p* value
**Mean Age – yrs**	63.0±10.8	62.8±10.9	0.96
**Male Gender**	23 (67.6)	17 (77.2)	0.43
**MELD**	8.7±4.5	10.6±4.8	0.14
**Child Pugh**	7.0±1.8	7.7±2.0	0.17
**Albumine – g/dl**	3.3±0.6	3.1±0.4	0.35
**Bilirubin – mg/dl**	2.0±3.1	2.3±1.8	0.67
**INR**	1.2±0.2	1.3±0.2	0.20
**Hepatic Encephalopathy**			
** **Grade 1Grade 2Grade 3	33 (97.0)1 (3.0)0 (0.0)	19 (86.4)3 (13.6)0 (0.0)	0.12
**Platelets – 10^3^/mmc**	109.0±79.0	64.9±22.9	0.01
**Aspartate aminotransferase – IU**	78.2±85.4	78.6±71.2	0.98
**Alanine aminotransferase – IU**	58.3±49.3	77.2±122.8	0.43
**Creatinine – mg/dl**	0.9±0.3	0.9±0.3	0.78
**Spleen Diameter – cm**	14.8±4.5	16.8±2.7	0.08
**Portal vein Diameter – mm**	12.2±2.3	13.2±2.7	0.16
**Ascites**	17 (50.0)	13 (59.1)	0.50
**Hepatocellular Carcinoma**	12 (35.3)	13 (59.1)	0.08
**Arterial Hypertension**	9 (26.4)	2 (9.1)	0.11
**Type 2 Diabetes**	8 (23.5)	10 (45.4)	0.09
**Beta-blocker therapy**	7 (20.5)	17 (77.3)	<0.001
NT pro BNP – pg/ml*	302.8±338.5	385.9±329.4	0.39
**Mean septal/lateral e**'	0.09±0.02	0.11±0.03	0.08
**MV E Vel – m/s**	0.66±0.19	0.91±0.23	<0.001
**E/e**' **ratio**	7.7±2.9	8.7±3.5	0.31
**Left Atrium – ml**	57.7±26.8	67.3±23.8	0.18
**MV E/A ratio**	0.89±0.33	1.21±0.46	0.006
**LVEDV MOD a4C – ml**	86.8±39.2	95.7±23.4	0.35
**EF – %**	63.0±5.9	62.1±8.4	0.63
**PAPs – mmHg**	29.3±5.2	32.0±4.6	0.12
**LVd Mass Ind (ASE) – g/m2**	88.7±24.6	79.0±23.6	0.20

## Discussion

In cirrhotic patients, NT-pro-BNP serum levels as well as echocardiographic parameters such as left atrial volumes and ejection fraction are significantly altered. There is a significant association between NT-pro-BNP serum levels, parameter of kidney and liver function, and atrial volumes. We also observed that cirrhotic patients with ascites, compared to their counterpart without, had higher NT-pro-BNP serum levels and accordingly larger atrial volumes, and a paradoxical significant increase in E/A ratio also observed in patients with large esophageal varices.

In an attempt to investigate the spectrum of cardiac abnormalities involving cirrhotic patients, we found that atrial volumes, ejection fraction, and partially left ventricular mass and PAPs are significantly altered, as compared to the hypertensive controls. These findings could be expression of the physio-pathological events that lead to the hyperdynamic syndrome, typical of cirrhosis, and characterized by increased heart rate and cardiac output and decreased systemic vascular resistance with normal or low blood arterial pressure [22]. Along this line we also observed higher NT-pro-BNP serum levels in cirrhotic compared to controls, this issue being partly explained by the relationship between the hormone and atrial volumes. Levels of NT pro-BNP may be related to large atrial volumes as a consequence of heart wall injury, and myocardiocyte stretching is one of the main factor stimulating natriuretic hormones production [9]. Accordingly, our data confirm the hypothesis already reported by Henriksen et al [12], that NT pro-BNP levels could be a marker of cardiovascular diastolic dysfunction in patients with end stage liver disease, acting as mediator of splanchnic vasodilatation in liver cirrhosis [8], [9]. In fact, in our cirrhotic patients NT pro-BNP levels were related not only to large atrial volumes, but also to indexes of liver and kidney function.

Another relevant issue of our study, is that high levels of NT pro-BNP are increased in the presence of ascites as compared to cirrhotic patients without, probably as a result of increased fluid retention. We also observed a trend for larger atrial volumes in ascitic versus no ascitic patients. Overall these data suggest that NT pro-BNP is a marker of cardiac subclinical dysfunction participating to liver decompensation. Further studies are needed to assess the role of NT pro-BNP as a predictor of decompensation and prognosis in cirrhotic patients.

In this study we also observed that patients with more advanced liver disease, i.e. those with large varices or with ascites, had a higher E/A ratio, and a relatively “better” diastolic cardiac function. This finding is explained by the fact that these patients usually need of periodic paracentesis and albumin infusion, determining an improvement of E/A ratio. In fact, it has been already described that after paracentesis and albumin infusion cirrhotic patients show an upgrading of diastolic dysfunction as demonstrated by an increase of E/A ratio [22]–[23]. Further, it seems that albumin have a direct effect on the myocardium, as well [24]–[26]. In addition, all patients with large esophageal varices are on therapy with beta-blockers for prophylaxis of the variceal bleeding, and these drugs, showing a vasoconstrictor effect on splanchnic compartment, determining an increase of cardiac pre-load with a more efficacy of the cardiac pump, and the disappearing of the diastolic dysfunction with a normalization of E/A ratio [27]. We also found an inverse association between large esophageal varices and platelet count, being these last a surrogate marker of portal hypertension as previously reported by Giannini et al [28].

The main limitation of this study, besides the unavoidable clinical heterogeneity and the relatively small number of patients, lies in its cross-sectional nature, unable to identify pathogenic mechanisms(s) linking cardiac dysfunction, NT pro BNP serum levels, and severity of liver disease. In addition the use of diuretics and beta-blockers in cirrhotics and of hypertensive patients as controls could also bias our results, underestimating the cardiac alterations of cirrhotic patients.

In conclusion, our study showed that cirrhotic patients have echocardiographic (larger atrial volumes) and biochemical (higher NT pro BNP serum levels) alterations expression of cardiac dysfunction, leading as spectator or actor to liver decompensation and ascites development. While further physio-pathological studies should be performed in order to determine whether the high levels of NT-pro BNP are a consequence of the hyperdynamic syndrome and volume overload on heart chambers. It is clinically relevant that NT-pro-BNP plasma levels are increased proportionally to the severity of cirrhosis. In fact, advanced cirrhosis and high NT-pro-BNP levels are significantly associated to increased LAV, and to signs of cardiac diastolic dysfunction, which characterize morpho-functional changes usually found in end stage liver disease. The correlation between NT pro-BNP levels and cirrhosis stage could eventually, if confirmed by longitudinal studies, prove useful as a prognostic indicators of *early decompensation* of cirrhosis.

## Supporting Information

Table S1 Demographic, clinical and laboratory features of 58 cirrhotic patients and 28 sex and age matched controls.
(DOC)
