# Supplementary material for: NT Pro BNP Plasma Level and Atrial Volume Are Linked to the Severity of Liver Cirrhosis
Source: PLoS One. 2013 Aug 5;8(8):e68364. doi: 10.1371/journal.pone.0068364 (PMC3734231; doi:10.1371/journal.pone.0068364)
Supplement: Table S1 — Demographic, clinical and laboratory features of 58 cirrhotic patients and 28 sex and age matched controls. (DOC) [file pone.0068364.s001.doc]

**Supplemental Table 1. Demographic, clinical and laboratory features of 58 cirrhotic patients and 28 sex and age matched controls.**

| **Variable** | **Cirrhotic Patients**  (n=58) | **Control Population**  (n=28) | **p value** |
| --- | --- | --- | --- |
| **Mean Age** – **yrs** | 62.9 ± 10.6 | 60.6 ± 8.4 | 0.31 |
| **Male Gender** | 42 (72.4) | 20 (71%) | 0.92 |
| **Etiology of Cirrhosis** |  | - | - |
| HCV | 34 (58.6) | - | - |
| HBV | 8 (13.8) | - | - |
| Alcohol | 2 (3,4) | - | - |
| Criptogenic | 6 (10.4) | - | - |
| Post-NASH | 8 (13.8) | - | - |
| **CHILD PUGH** | 7.4 ± 2.0 | - | - |
| A5-A6 | 24 (41.5) | - | - |
| B7-B9 | 23 (39.6) | - | - |
| ≥C10 | 11 (18.9) | - | - |
| **MELD** | 9.7 ± 4.9 | - | - |
| **Albumin – g/dl** | 3.2 ± 0.5 | 4.2 ± 0.3 | <0.001 |
| **Bilirubin – mg/dl** | 2.2 ± 2.8 | 0.6 ± 0.4 | 0.005 |
| **INR** | 1.3 ± 0.3 | 0.9 ± 0.1 | <0.001 |
| **Ascites** | 32 (55.2) | - | - |
| **Hepatic Hencephalopathy** |  | - | - |
| Grade 1 | 54 (93.1) | - | - |
| Grade 2 | 4 (6.9) | - | - |
| Grade 3 | 0 (0) |  |  |
| **Platelets - 10^3^/mmc** | 90.6 ± 64.4 | 256.5 ± 98.7 | <0.001 |
| **Aspartate aminotransferase – IU** | 77.5 ± 78.7 | 21.0 ± 9.6 | <0.001 |
| **Alanine aminotransferase – IU** | 65.0 ± 86.0 | 27.1 ± 2.9 | <0.001 |
| **Creatinine – mg/dl** | 0.9 ± 0.3 | 0.8 ± 0.3 | 0.59 |
| **Spleen Diameter - cm** | 15.7 ± 3.8 | - | - |
| **Portal vein Diameter - mm** | 12.5 ± 2.5 | - | - |
| **Oesophageal Varices** |  | - | - |
| Absent | 10 (17.2) | - | - |
| F1 | 24 (41.4) | - | - |
| F2-F3 | 22 (37.9) | - | - |
| Unknown | 2 (1.2) | - | - |
| **Stage of Cirrhosis** |  | - | - |
| No varices, no ascites | 4 (6.9) | - | - |
| Varices, no ascites | 22 (37.9) | - | - |
| Ascites | 32 (55.2) | - | - |
| **Hepatocellular Carcinoma** | 32 (55.1) | - | - |
| **Arterial Hypertension** | 11 (18.9) | 28 (100) | <0.001 |
| **Type 2 Diabetes** | 18 (31.0) | 8 (28) |  |
| **Beta-blocker therapy** | 25 (43.1) | 0 |  |
